# Clinical Evaluation of a Novel CGM-Informed Bolus Calculator with Automatic Glucose Trend Adjustment

**DOI:** 10.1089/dia.2021.0140

**Published:** 2022-01-05

**Authors:** Jordan E. Pinsker, Mei Mei Church, Sue A. Brown, Mary K. Voelmle, Bruce W. Bode, Brooke Narron, Lauren M. Huyett, Joon Bok Lee, Jason O'Connor, Eric Benjamin, Bonnie Dumais, Trang T. Ly

**Affiliations:** ^1^ Sansum Diabetes Research Institute, Santa Barbara, California, USA.; ^2^ Division of Endocrinology, Center for Diabetes Technology, University of Virginia, Charlottesville, Virginia, USA.; ^3^ Atlanta Diabetes Associates, Atlanta, Georgia, USA.; ^4^ Insulet Corporation, Acton, Massachusetts, USA.; Results of this study were presented in abstract form at the 14th International Conference on Advanced Technologies & Treatments for Diabetes, June 2021.

**Keywords:** Continuous glucose monitoring, Bolus calculator, Hypoglycemia, Omnipod, Type 1 diabetes

## Abstract

***Background:*** Expert opinion guidelines and limited data from clinical trials recommend adjustment to bolus insulin doses based on continuous glucose monitor (CGM) trend data, yet minimal evidence exists to support this approach. We performed a clinical evaluation of a novel CGM-informed bolus calculator (CIBC) with automatic insulin bolus dose adjustment based on CGM trend used with sensor-augmented pump therapy.

***Materials and Methods:*** In this multicenter, outpatient study, participants 6–70 years of age with type 1 diabetes (T1D) used the Omnipod^®^ 5 System in Manual Mode, first for 7 days without a connected CGM (standard bolus calculator, SBC, phase 1) and then for 7 days with a connected CGM using the CIBC (CIBC phase 2). The integrated bolus calculator used stored pump settings plus user-estimated meal size and/or either a manually entered capillary glucose value (SBC phase) or an imported current CGM value and trend (CIBC phase) to recommend a bolus amount. The CIBC automatically increased or decreased the suggested bolus amount based on the CGM trend.

***Results:*** Twenty-five participants, (mean ± standard deviation) 27 ± 15 years of age, with T1D duration 12 ± 9 years and A1C 7.0% ± 0.9% completed the study. There were significantly fewer sensor readings <70 mg/dL 4 h postbolus with the CIBC compared to the SBC (2.1% ± 2.0% vs. 2.8 ± 2.7, *P* = 0.03), while percent of sensor readings >180 and 70–180 mg/dL remained the same. There was no difference in insulin use or number of boluses given between the two phases.

***Conclusion:*** The CIBC was safe when used with the Omnipod 5 System in Manual Mode, with fewer hypoglycemic readings in the postbolus period compared to the SBC. This trial was registered at ClinicalTrials.gov (NCT04320069).

## Introduction

Expert opinion guidelines and limited data from clinical trials recommend adjustment to bolus insulin doses based on continuous glucose monitoring (CGM) trend data.^1–5^ Variation exists in these recommendations,^6^ and to date, individuals have been required to manually calculate adjusted doses, leading to extensive discrepancies between published recommendations versus actual doses delivered.^7,8^

Although bolus calculators have existed in various forms since 2003,^9^ until now, bolus calculators in insulin pumps only allowed users to enter a glucose value as a static point. While current bolus calculators in insulin pumps do account for insulin on board (IOB), they do not provide a means to automatically incorporate CGM trend calculations into their bolus recommendations. This study evaluated a novel CGM-informed bolus calculator (CIBC) with automatic glucose trend adjustment used with sensor-augmented pump therapy, which is built into the Omnipod^®^ 5 Automated Insulin Delivery System (Insulet Corporation, Acton, MA).

To date, the CIBC has been used safely by hundreds of clinical study participants using the Omnipod 5 System in Automated Mode^10,11^ (also known as hybrid closed-loop control or automated insulin delivery); however, it has not been evaluated specifically when used with Manual Mode (open-loop control). In addition, there is a lack of data comparing the performance of the CIBC with that of a standard bolus calculator (SBC). To address these needs, we compared 7 days of use in Manual Mode without a connected CGM (SBC) to 7 days of use in Manual Mode with a connected CGM using the CIBC, evaluating the safety and effectiveness of the Omnipod 5 CIBC in individuals with type 1 diabetes (T1D).

## Materials and Methods

### Study design

This investigation was a single-arm, multi-center, prospective study performed at three clinical sites (Sansum Diabetes Research Institute, Atlanta Diabetes Associates, and University of Virginia). The study schedule consisted of two outpatient phases. In phase 1, participants completed 7 days of Omnipod 5 use in Manual Mode without a connected CGM using manual entry of capillary blood glucose (BG) values into the bolus calculator. Participants wore a CGM (Dexcom G6; Dexcom, Inc., San Diego, CA), but it was not connected to the Omnipod 5 System. Boluses were calculated by the SBC using stored pump settings plus user-estimated grams of carbohydrates in the meal and a manually entered capillary BG value. Participants received a follow-up phone call on day 3 of phase 1 to assure they were correctly entering capillary BG measurements into the system before each bolus.

Participants then completed 7 days of Omnipod 5 use in Manual Mode with a connected CGM using the CIBC to deliver boluses (phase 2). In phase 2, boluses were calculated by the CIBC using stored pump settings plus user-estimated grams of carbohydrates in the meal and an imported current CGM value and CGM trend. Participants also received a follow-up phone call on day 3 of phase 2 to assure they were instructing the system to use the CIBC for each bolus.

Key eligibility criteria included age of 6–70 years with T1D for at least 6 months. Key exclusion criteria included history of severe hypoglycemia or diabetic ketoacidosis in the past 6 months, pregnancy or lactation, or chronic disease such as kidney disease, adrenal insufficiency, or need for steroid use, which could have affected the study results or put the participants at risk. All participants were required to use U-100 insulin intended for use in the study device. Use of any noninsulin glucose-lowering agent other than metformin (e.g., GLP1 agonist, SGLT2 inhibitor, DPP-4 inhibitor, pramlintide) was prohibited.

The protocol was approved by the Western Institutional Review Board. Informed consent was obtained from participants 18 years of age and older. For participants younger than 18 years, assent and consent were obtained from participants and their parents or guardians, respectively, according to state requirements. The United States Food and Drug Administration approved an investigational device exemption. This trial was registered at ClinicalTrials.gov (NCT04320069).

### CGM-informed bolus calculator

The CIBC is a tool that can be used to calculate and deliver correction and meal boluses in the Omnipod 5 System both in Automated and Manual Modes. The design of the CIBC and its integration into the Omnipod 5 System have been previously described.^10^ A key novel feature of the CIBC is that it automatically incorporates both CGM value and trend information when determining bolus amounts.

**FIG. 1.**
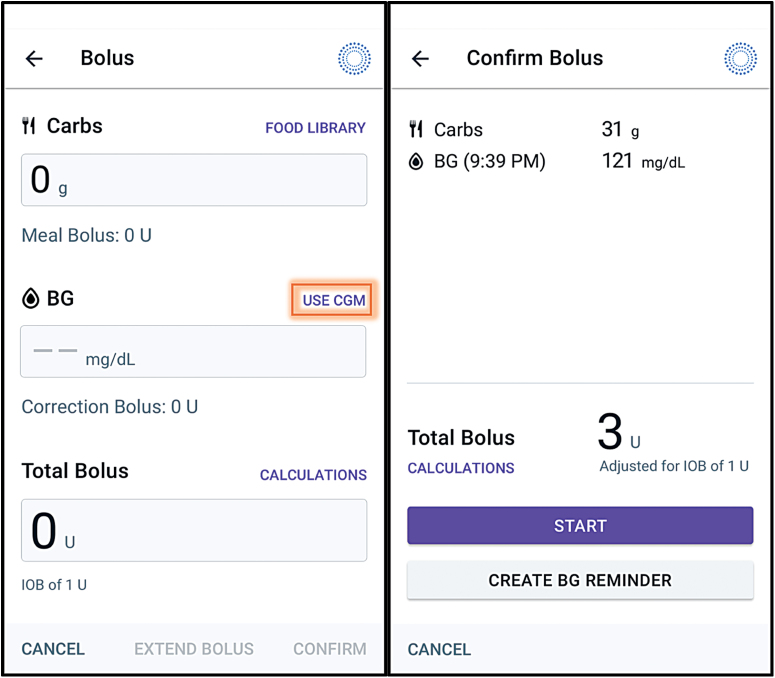
Omnipod 5 application displaying (left) Bolus Calculator screen where users may press the *USE CGM* button (highlighted with orange box) and (right) Confirm Bolus screen, which calculates the total bolus units needed for the BG value and trend imported from the CGM, as well as any entered carbohydrates. BG, blood glucose; CGM, continuous glucose monitor. Color images are available online.

To activate the CIBC, the user selects the *USE CGM* button on the bolus calculator screen, which automatically imports the current CGM value and trend (Fig. 1). If the user instead manually enters a glucose value or leaves the BG field blank, the SBC will be used. Both the SBC and the CIBC begin with the same calculation: the initial bolus recommendation is determined based on the user's personalized bolus settings (insulin to carbohydrate ratio, correction factor, and target glucose), the current glucose value, the amount of carbohydrates entered in grams (if any) and any pre-existing IOB. The method for adjusting for IOB is described in more detail below. When using the SBC with a manual glucose value or a blank BG field, the calculation stops here, and this is the final bolus amount recommended to the user.

**FIG. 2.**
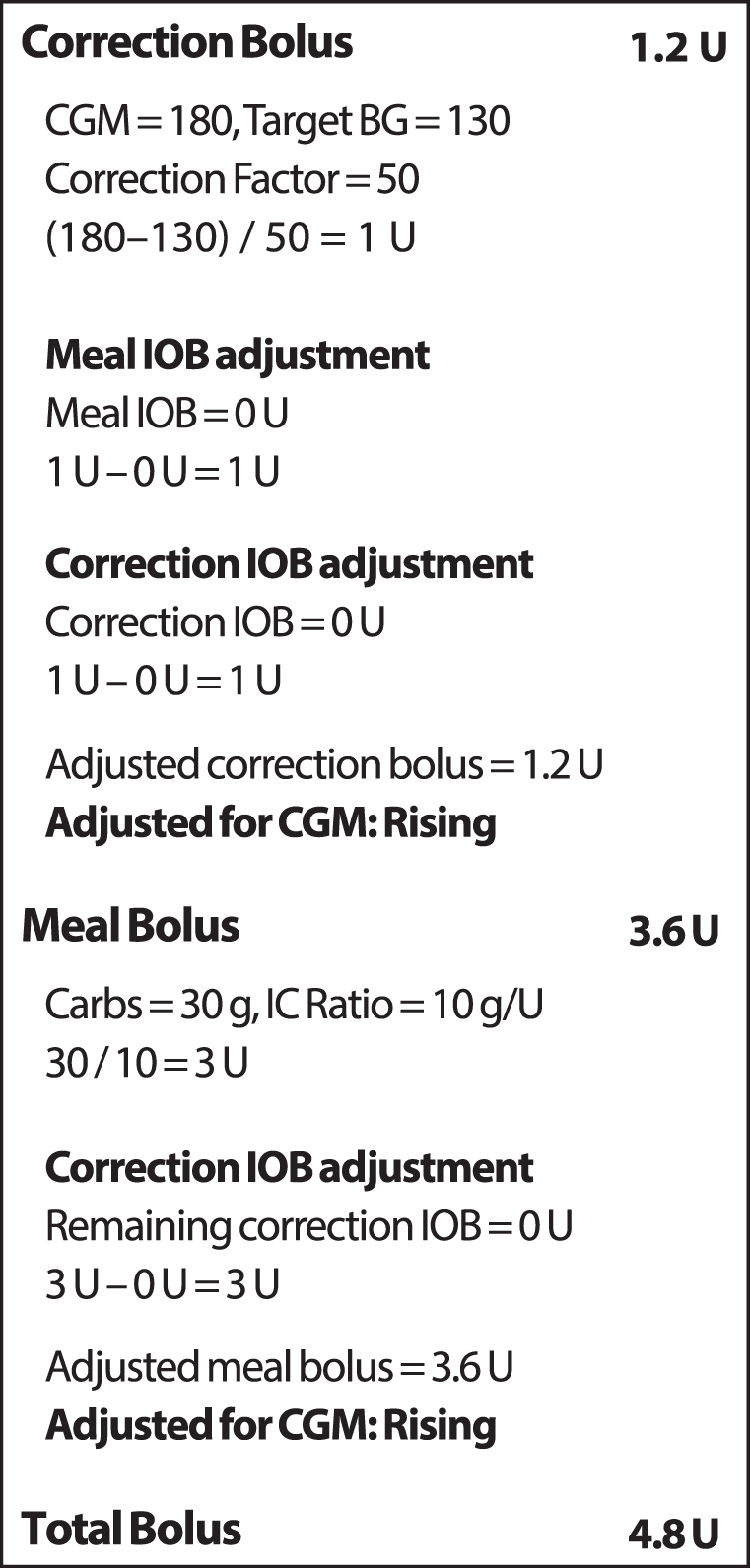
Example calculation with the Omnipod 5 System CGM-informed bolus calculator. In this scenario, the current CGM value is 180 mg/dL with a rising trend, the target BG is 130 mg/dL, the correction factor is 50 mg/dL per unit, and the insulin to carbohydrate ratio is 10 g per unit. There is no IOB. The correction bolus is initially calculated as 1 U. First, the Meal IOB and then the Correction IOB are subtracted from this amount; however, in this example, both IOB amounts are zero so the correction bolus is unchanged at 1 U. The correction bolus is then automatically adjusted from 1 to 1.2 U based on the rising glucose trend. The meal bolus is initially calculated as 3 U. Any remaining Correction IOB is then subtracted; however, in this example, there is no IOB so the meal bolus amount remains at 3 U. The meal bolus is then automatically adjusted from 3 to 3.6 U based on the rising glucose trend. The total bolus is the correction bolus plus the meal bolus (1.2 U plus 3.6 U) for a total of 4.8 U. If using the standard bolus calculator with the same conditions, the total bolus amount would have been determined from the correction and meal bolus amounts before trend adjustment (1 U plus 3 U) for a total of 4 U. Therefore, an additional 0.8 U has been recommended to account for the rising glucose trend. IOB, insulin on board.

When using the CIBC, the bolus recommendation is further modified based on the magnitude and direction of the glucose trend as measured by the CGM. Part of this adjustment is dependent on proprietary factors that are directly proportional to the magnitude of the trend. The adjustment also considers the projected glucose concentrations based on the given trend for further refinement. The total recommended bolus amount may be increased by up to 30% when the CGM trend shows that the glucose level is increasing or decreased by up to 100% (zero bolus) when the CGM trend shows that the glucose level is decreasing. These adjustments to recommended bolus doses are available in both Automated and Manual Modes. Similar to all current insulin pumps, users are able to adjust the recommended insulin bolus amount before delivery. An example bolus calculation is shown in Figure 2.

IOB accounts for insulin that has previously been delivered and is still active in the body. The IOB remaining from past boluses is calculated using the duration of insulin action parameter entered into the pump, which is customizable and impacts user-initiated bolus delivery. The system accounts for IOB from past meal boluses (Meal IOB) separately from IOB from past correction boluses (Correction IOB). Correction IOB also includes IOB from past basal delivery above the user's anticipated basal need, such as from past automated insulin delivery above basal in Automated Mode. When delivering a new bolus through the CIBC or SBC, first, any existing Meal IOB is subtracted from the correction portion of the new bolus. Then, any existing Correction IOB is subtracted from the correction portion of the new bolus. Any remaining Correction IOB is then subtracted from the meal portion of the new bolus. Meal IOB is never subtracted from the meal portion of a new bolus. This process is illustrated in Figure 2.

### Outcome measures

The primary objective of this study was to evaluate the safety of the CIBC during Manual Mode using glucose metrics of percentage of time with sensor glucose <70 and >180 mg/dL during the 4-h postbolus period from phase 1 compared to phase 2.

Secondary objectives were to evaluate the effectiveness of the CIBC using glucose metrics from phase 1 compared to phase 2. Additional glucose metrics during the 4-h postbolus period compared from phase 1 to phase 2 included mean sensor glucose and percent of time with sensor glucose <54, ≥250, ≥300, and 70–180 mg/dL.

Comparisons of glucose metrics from phase 1 to phase 2 were also assessed during the day (6 AM up to 12 AM), overnight (12 AM up to 6 AM), and overall, and included the following: mean, standard deviation, and coefficient of variation of sensor glucose, and percent of time with sensor glucose <54, <70, >180, ≥250, ≥300, 70–180, and 70–140 mg/dL.

Total daily insulin use, basal and bolus insulin use, and number of boluses per day were also compared between the two phases.

### Statistical analysis

The sample size was not hypothesis driven and was chosen to provide adequate information on the device's safety and performance. The primary and secondary endpoints were reported using a modified intention-to-treat analysis set, which included all participants who successfully entered phase 2 of the study. Values are represented as mean ± standard deviation unless otherwise noted. The difference in outcomes between phase 1 and phase 2 was assessed using two-sided paired *t*-tests. All *P*-values were considered significant at a two-sided level of 0.05. Analysis was performed using SAS version 9.4.

## Results

Twenty-five participants 6–70 years of age diagnosed with T1D for at least 6 months and meeting the eligibility criteria were enrolled across three clinical sites. The mean duration of phase 1 was 7.0 days (range 6.7–9.4 days), and the mean duration of phase 2 was 6.6 days (range 4.7–10.0 days). The mean number of days the Omnipod 5 System was connected to the CGM in phase 2 was 6.3 days (range 4.7–8.2 days) with a cumulative experience of 157.3 days of CGM connection.

Due to the current COVID-19 pandemic, participants were recruited from the Omnipod 5 Pivotal Study (NCT04196140) before their recommencement of the pivotal study following a study pause to fix a software anomaly. This recruitment allowed for minimizing participant contacts during the pandemic, while limiting the need for in-person training as participants were already familiar with the system. Participant demographics are summarized in Table 1. All participants completed the scheduled follow-ups; there were no early withdrawals or missed study visits.

**Table 1.** Participant Demographics and Baseline Characteristics (Mean ± Standard Deviation)

Characteristic	Participants (*N* = 25)
Age (years), range	26.9 ± 15.4, range 7.6–63.0
Gender (*n*)	
Male	9
Female	16
Weight (kg)	66.7 ± 27.6
Body mass index (kg/m^2^)	23.8 ± 6.2
HbA1c (%)	7.0 ± 0.9
Duration of diabetes (years)	12.2 ± 9.4

The primary objective of this study was to evaluate the safety of the CIBC using glucose metrics of percent time <70 mg/dL and percent time >180 mg/dL during the 4-h postbolus period from phase 1 compared to phase 2. Sensor glucose metrics in the 4-h postbolus period are summarized in Table 2. Percent time sensor glucose levels were <70 mg/dL decreased from phase 1 to phase 2 by 0.6% (phase 1, 2.8%, phase 2, 2.1%, *P* = 0.03). Percent time sensor glucose levels were >180 mg/dL differed by 1.9%, which was not statistically significant (phase 1, 32.1% and phase 2, 34.0%, *P* = 0.4). Percent time sensor glucose levels were in the target range of 70–180 mg/dL was 65.1% during phase 1 compared to 63.8% during phase 2 (*P* = 0.6).

**Table 2.** Comparison of Sensor Glucose Metrics Between Phase 1 (Standard Bolus Calculator) and Phase 2 (Continuous Glucose Monitor-Informed Bolus Calculator) in the 4-H Postbolus Period for *n* = 25 Participants

Sensor glucose metric	Phase 1	Phase 2	Difference	*P*
Primary outcomes				
Percent time <70 mg/dL	2.8 (2.7)	2.1 (2.0)	−0.6 (1.4)	0.03^*^
Percent time >180 mg/dL	32.1 (15.7)	34.0 (16.0)	1.9 (11.4)	0.4
Secondary outcomes				
Mean CGM glucose (mg/dL)	158.7 (21.3)	163.3 (23.6)	4.6 (15.1)	0.14
Percent time, %				
70–180 mg/dL	65.1 (15.4)	63.8 (15.7)	−1.3 (11.2)	0.6
<54 mg/dL	0.5 (1.0)	0.3 (0.7)	−0.2 (0.6)	0.16
≥250 mg/dL	8.2 (6.9)	9.7 (10.3)	1.4 (6.1)	0.25
≥300 mg/dL	2.0 (2.6)	2.6 (3.7)	0.6 (3.2)	0.32

^*^ Significant with *P*-value <0.05; results are shown as mean (standard deviation).

CGM, continuous glucose monitor.

Additional sensor glucose metrics for the daytime (6 AM to <12 AM), nighttime (12 AM to <6 AM), and overall, in both phases are summarized in Table 3. The percentage of time sensor glucose levels were in target range 70–180 mg/dL was similar between phase 1 and phase 2 across the three time periods evaluated. During phase 1, percent time sensor glucose levels were 70–180 mg/dL during the day was 71.1% compared to 70.6% during phase 2 (*P* = 0.81). During the night, this measure was 70.3% and 70.4% for phase 1 and phase 2, respectively (*P* = 0.98). Results for overall (day and night) percent time 70–180 mg/dL were 70.9% for phase 1 and 70.6% for phase 2 (*P* = 0.86).

**Table 3.** Sensor Glucose Metrics Comparison Between Phase 1 (Standard Bolus Calculator) and Phase 2 (Continuous Glucose Monitor-Informed Bolus Calculator) for the Daytime (6 AM to <12 AM), Nighttime (12 AM to <6 AM), and Overall (24 H) for *n* = 25 Participants

Sensor glucose metric	Phase 1	Phase 2	Difference	*P*
Overall (24-h)				
Mean CGM glucose (mg/dL)	150.4 (18.9)	153.5 (20.3)	3.1 (15.0)	0.31
Standard deviation (mg/dL)	51.9 (12.1)	51.6 (12.8)	−0.3 (7.7)	0.86
Coefficient of variation (%)	34.3 (5.8)	33.4 (6.3)	−0.9 (4.3)	0.32
Percent time, %				
<54 mg/dL	0.4 (0.7)	0.4 (0.5)	−0.1 (0.5)	0.5
<70 mg/dL	2.9 (2.3)	2.7 (2.0)	−0.2 (1.7)	0.49
70–140 mg/dL	47.2 (13.8)	44.9 (14.8)	−2.3 (12.2)	0.36
70–180 mg/dL	70.9 (13.2)	70.6 (14.1)	−0.4 (10.6)	0.86
>180 mg/dL	26.2 (13.7)	26.8 (14.0)	0.6 (10.6)	0.78
≥250 mg/dL	6.3 (5.1)	7.3 (7.4)	1.0 (5.1)	0.33
≥300 mg/dL	1.4 (1.8)	1.8 (2.3)	0.4 (2.1)	0.34
Daytime (06:00–23:59)				
Mean CGM glucose (mg/dL)	150.1 (18.1)	152.8 (20.7)	2.8 (14.6)	0.36
Standard deviation (mg/dL)	52.1 (12.2)	51.4 (12.8)	−0.7 (6.3)	0.57
Coefficient of variation (%)	34.6 (6.6)	33.5 (6.7)	−1.1 (4.1)	0.19
Percent time, %				
<54 mg/dL	0.5 (0.8)	0.3 (0.7)	−0.2 (0.5)	0.13
<70 mg/dL	2.9 (2.7)	2.7 (2.2)	−0.3 (1.6)	0.36
70–140 mg/dL	47.4 (13.9)	45.8 (15.2)	−1.6 (12.6)	0.53
70–180 mg/dL	71.1 (13.4)	70.6 (14.3)	−0.5 (10.8)	0.81
>180 mg/dL	25.9 (13.5)	26.8 (14.2)	0.8 (10.7)	0.7
≥250 mg/dL	6.3 (4.9)	7.0 (8.3)	0.7 (5.3)	0.49
≥300 mg/dL	1.4 (1.9)	1.9 (2.8)	0.4 (2.1)	0.31
Nighttime (00:00–05:59)				
Mean CGM glucose (mg/dL)	151.4 (27.7)	155.6 (28.3)	4.2 (22.4)	0.36
Standard deviation (mg/dL)	47.3 (15.6)	47.2 (16.7)	−0.2 (15.5)	0.95
Coefficient of variation (%)	31.0 (7.1)	30.0 (8.0)	−1.0 (8.2)	0.56
Percent time, %				
<54 mg/dL	0.3 (0.9)	0.4 (0.7)	0.2 (1.1)	0.48
<70 mg/dL	2.8 (4.1)	2.7 (3.7)	−0.1 (4.2)	0.89
70–140 mg/dL	46.5 (18.1)	42.1 (18.5)	−4.4 (17.5)	0.22
70–180 mg/dL	70.3 (17.2)	70.4 (18.8)	0.1 (16.3)	0.98
>180 mg/dL	26.9 (18.9)	26.9 (19.0)	0.1 (15.8)	0.99
≥250 mg/dL	6.3 (8.3)	8.2 (11.2)	1.8 (10.2)	0.37
≥300 mg/dL	1.5 (3.4)	1.8 (3.5)	0.3 (3.9)	0.72

Results are shown as mean (standard deviation).

There were no significant differences in total daily insulin use, basal insulin use, or bolus insulin use between each phase (Table 4). The number of boluses per day, 6.4 ± 2.9 and 6.8 ± 2.9 with the SBC and CIBC, respectively, was also similar (*P* = 0.3).

**Table 4.** Insulin Requirement (U/kg) Comparison Between Phase 1 (Standard Bolus Calculator) and Phase 2 (Continuous Glucose Monitor-Informed Bolus Calculator) for *n* = 25 Participants

Insulin requirement (U/kg)	Phase 1	Phase 2	Difference	*P*
Total daily insulin	0.65 (0.32)	0.66 (0.34)	0.01 (0.08)	0.61
Total daily basal insulin	0.31 (0.14)	0.31 (0.14)	0.00 (0.01)	0.29
Total daily bolus insulin	0.34 (0.21)	0.34 (0.23)	0.01 (0.08)	0.72

Results are shown as mean (standard deviation).

The percentage of boluses delivered using the bolus calculator was median (interquartile range) 98% (96%, 100%) in phase 1 and 100% (99%, 100%) in phase 2. Furthermore, of all boluses delivered in each phase, 89% (71%, 98%) in phase 1 and 91% (73%, 98%) in phase 2 were delivered using the calculator recommendation without any adjustment by the user (*P* = 0.6 by Wilcoxon signed-rank test). In phase 2, 86% (68%, 92%) of the boluses delivered as per the calculator recommendation without user adjustment included the CGM value and trend as part of the calculation.

There were no unanticipated adverse device effects or serious adverse events related to use of the system. There was 1 serious adverse event of pyelonephritis, which was unrelated to the study procedures and device. There were no nonserious adverse events reported.

## Discussion

In this 14-day home use insulin pump study, we compared the safety and effectiveness of using static capillary glucose measurements in an SBC with that of a bolus calculator that incorporates CGM glucose values and trends (CIBC). The results showed that there were significantly fewer readings <70 mg/dL in the 4-h postbolus period when using the CIBC compared to the SBC, while all other glycemic metrics remained the same. This investigation is the first clinical study to perform a direct comparison of glycemic outcomes with and without the use of CGM trend in bolus calculations under otherwise identical conditions, with automatic calculations to include CGM trend information incorporated directly into an insulin pump. In fact, a significant strength of this study is that the two phases were both conducted at home without any additional training or education intervention that could influence results. There was no difference in the total daily insulin delivery or number of boluses between the two phases, which serves as another indication that eating behaviors, activity levels, and other treatment patterns were likely consistent between the two phases.

The use of CGM trend to adjust insulin bolus dosing seems intuitive, yet to date, no other insulin pump system has incorporated such a feature directly into the bolus calculator. Previous reports have examined the approach to manually adjusting insulin bolus doses based on CGM trend.^5,12,13^ To date, only two clinical trials exist to support this approach, both focusing solely on the pediatric age group. One study used the now-discontinued Abbott Freestyle Navigator CGM system with manual calculations for multiple daily injection users, where a 10%–20% dose adjustment was used.^5^ In the other, participants at a diabetes camp were instructed to adjust bolus doses based on predicted glucose values using the trend from the Dexcom G5 CGM.^1^ While these studies showed promising results, it is difficult to draw any conclusion about the definitive effect of the trend adjustment alone.

In 2017, the Endocrine Society published a pair of expert panel recommendation articles on adjusting insulin bolus doses based on the CGM trend from the Dexcom G5 in both adults and children.^3,4^ Kudva et al. further elaborated on these guidelines, discussing their use with the FreeStyle Libre system.^2^ Despite this guidance, it has been reported that CGM users often make much larger changes than recommended to insulin doses when trying to adjust for sensor glucose trends,^7,8^ demonstrating how challenging it is to manually consider CGM trend input when adjusting a bolus insulin dose. The lack of consistency among published guidelines, with some studies recommending a 10%–20% dose adjustment, others endorsing increasing or decreasing the sensor value used for the bolus calculation by 25–100 mg/dL, and still others suggesting outright increasing or decreasing the overall insulin dose by 1 to 4.5 U,^6^ makes it difficult for an individual to decide how best to use their CGM data. Since most insulin pumps can now be connected to a CGM, bolus calculators have the potential to perform this calculation automatically and consistently, reducing the burden on the user whether using automated or manual insulin delivery.

In the past, it has been suggested that bolus calculator use could improve outcomes when used consistently by people with T1D,^14,15^ but a recent well-designed 10-month randomized crossover study using an automated bolus calculator device failed to show improvements in HbA1c or quality of life.^16^ Although this study was conducted using a simpler bolus calculator based on static BG readings for multiple daily injection users, the requirement for the user to enter insulin doses and BG readings manually for correct usage likely affected outcomes. When designing new diabetes technology or treatment methods, it is important to consider the ease of use and burden placed on the user for correct use, which will influence the success of the treatment.

Our results indicated that the CIBC was safe when used in Manual Mode, with significantly fewer hypoglycemic readings in the 4-h postbolus period compared to the SBC. No other significant change in sensor glucose metrics was noted, including the overall, daytime and nighttime analysis. This result was not surprising, as the primary aim was to establish the safety of this system configuration, as it is possible a subset of future users of the system will elect to use the system in Manual Mode, but still use the features of the CIBC.

Additional studies on advanced bolus calculator features have recently been reported. Fabris et al. showed a reduction in postprandial hypoglycemia, without increasing hyperglycemia, using a smart bolus calculator informed by real-time insulin sensitivity assessments following aerobic exercise.^17^ They also reported a reduction in hypoglycemia rescue treatments. Adjustments to bolus calculations have also been incorporated into decision support systems that performed automated insulin dose titration, conducted bolus calculations, and gave carbohydrate treatment recommendations.^18^ In the research setting, some systems such as the interoperable artificial pancreas system automatically reduced the recommended insulin bolus dose by 20% if the current CGM value was below a certain threshold.^19^ These studies show the potential for further advancements to insulin bolus calculators that could be integrated into connected insulin pumps.

We recognize limitations of this analysis. This study was of a relatively short duration with only 25 participants, potentially allowing for a type II error. With a larger cohort and longer use of the system, it may be possible to see further significant findings. Because of the small sample size, we could not compare differences between children, adolescents, and adults.

In addition, bolus recommendations were adjustable by the user, potentially reducing the effect of the recommendations if bolus doses were manually adjusted; however, our results show that most boluses were delivered according to bolus calculator recommendations in both phases, and the percentage of boluses following the calculator recommendation was similar in both phases. The study cohort achieved excellent control during both phases of the study, with the average time in range meeting the international consensus target of >70%.^20^ While the CIBC primarily affected hypoglycemia outcomes in this population, it is plausible that the CIBC could have a greater impact on time in range or time in hyperglycemia for those who have more difficulty achieving optimal glycemic control with an SBC. It is also possible that results may be different with Automated Mode active, as this study only evaluated use of CIBC in Manual Mode. Finally, we did not prescribe activity or meals, so there could have been differences in activity levels or meal boluses between the two phases, although the overall amount of insulin delivered, and number of boluses given did not differ.

## Conclusions

We conclude that the CIBC was safe when used in Manual Mode, with fewer hypoglycemic CGM readings in the postbolus period compared to the SBC. The CIBC, fully integrated into the Omnipod 5 System, is also available for use with Automated Mode, where its use was included during the pivotal study of the system.^10,11^
